# Challenges in schizoaffetive disorder therapeutic – a case report of a patient with hiperprolactinemia

**DOI:** 10.1192/j.eurpsy.2021.2107

**Published:** 2021-08-13

**Authors:** D. Rodrigues, D. Jeremias, C. Laginhas, A. Sequeira

**Affiliations:** Psychiatry Department, Ocidental Lisbon Hospital Center, Lisboa, Portugal

**Keywords:** schizoaffective disorder, prolactin, antipsychotic, psychosis

## Abstract

**Introduction:**

The only FDA approval therapeutic for schizoaffective disorder is paliperidone. Hiperprolactinemia is one of the most frequent side effects induced by first generation antipsychotics (FGA) or by second generation antipsychotic (SGA), such as risperidone and paliperidone. Prolactin related symptoms (PRS) include amenorrhea, galactorrhea, gynecomastia and fluctuations in psychotic symptoms.

**Objectives:**

To report the case of a patient with schizoaffective disorder difficult to manage due to symptom resistance and PRS, that improved symptomatology when prolactin serum levels were reduced.

**Methods:**

Clinical-demographic data collected by clinical interview and clinical process consultation. Non-systematic literature review, searching “psychosis”; “prolactin”; “antipsychotic”; “schizoaffective disorder” on Pubmed database.

**Results:**

We report the case of 33 years-old female, admitted to our psychiatry inpatient unit for persecutory delusions, loosening of association, auditory hallucinations, and irritability with functional impairment. Symptoms began 13 years before. She was medicated with paliperidone 100mg IM monthly, lithium 800mg daily and clozapine 225mg daily. When admitted she wasn’t adhering to oral medication. On physical examination presented some PRS. The serum presented hyperprolactinemia and lithium in non-therapeutic levels. Initially was re-introduced the previous therapeutic without improval. It was made a therapeutic switch to associate aripiprazole 400mg IM monthly and clozapine 225mg daily, and lithium 800mg daily resulting in prolactine normalization and subsequent improval of psychotic symptoms previously presented.

**Conclusions:**

This case reports challenges in management of patients diagnosed with Schizoaffetive Disorder due to therapeutic refractoriness and side effects. PRS can be ruling, therefore impacting therapeutic choices. We propose a possible role of combination of clozapine and aripiprazole in this scenario.

**Disclosure:**

No significant relationships.

